# Emerging Interventions to Improve Health Outcomes for People Aging With HIV: Protocol for a Mixed Methods Implementation Science Evaluation

**DOI:** 10.2196/72471

**Published:** 2025-11-07

**Authors:** Richard Dunville, Sarah Hodge, Shannon TenBroeck, Alexis Marbach, Christopher La Rose, Joanne Hsu, Tracy McClair, Demetrios Psihopaidas, Aging with HIV Study Group

**Affiliations:** 1 Department of Public Health NORC at the University of Chicago Chicago, IL United States; 2 Health Resources and Services Administration Rockville, MD United States; 3 See Acknowledgments

**Keywords:** implementation science, evaluation, HIV and aging, geriatric care, emerging interventions

## Abstract

**Background:**

In 2022, 54% of people with HIV were aged 50 years and older; however, clinical care for HIV in the United States often falls short of comprehensively integrating care for aging-related conditions. In response, the Health Resources and Services Administration HIV/AIDS Bureau Ryan White HIV/AIDS Program funded a new initiative comprising 10 demonstration sites to test emerging interventions to support people aging with HIV, as well as a capacity-building provider and an evaluation provider. NORC at the University of Chicago received an award for the evaluation provider.

**Objective:**

This protocol aimed to describe the application of the Health Resources and Services Administration HIV/AIDS Bureau implementation science (IS) framework to a multisite evaluation, a related evaluation protocol, the technical assistance provided to support the evaluation, and the initiative’s dissemination plan.

**Methods:**

Using a theory-based approach, NORC developed a mixed methods evaluation plan using an IS hybrid type 2 study with two main aims: (1) to describe implementation outcomes and (2) to assess client-level outcomes. Implementation outcomes were assessed at the organizational level using tools including a survey of site characteristics, key informant interviews, and documentation of monthly monitoring calls and costs. Client-level outcomes were assessed through a survey and a medical chart abstraction tool. NORC also collected data on the sites’ engagement with the capacity-building provider and their satisfaction with the services provided.

**Results:**

The evaluation was funded in August 2022. Organizational-level data collection began upon institutional review board approval in April 2023. All sites were enrolling clients in the intervention and evaluation by September 2023, and 626 clients enrolled by December 2023. Data collection is expected to continue through December 2024. Analysis of the baseline results is currently underway, and comprehensive findings are expected by late 2025.

**Conclusions:**

To the best of our knowledge, this is the first national study to evaluate emerging clinical interventions for people aging with HIV using an IS framework. The findings will build an evidence base for advancing HIV clinical care to meet the needs of the aging population.

**International Registered Report Identifier (IRRID):**

DERR1-10.2196/72471

## Introduction

### Background

Advances in HIV treatment have yielded profound improvements in life expectancy and quality of life among people with HIV. More than half of the people with HIV in the United States are now aged 50 years or older [1]. Health Resources and Services Administration (HRSA) HIV/AIDS Bureau (HAB) Ryan White HIV/AIDS Program clients are increasingly older as well. In 2022, 48.2% were aged 50 years and older and 24% were aged 60 years and older [2]. Relative to their younger peers, older adults with HIV are more likely to be retained in care and achieve viral suppression [1], although not all groups benefit equally, and certain populations continue to experience lower viral suppression rates [2].

Current comprehensive HIV care programs often fall short of meeting the variety of co-occurring age-related issues experienced by people aging with HIV. These include comorbidities associated with aging, as people aging with HIV are disproportionately diagnosed with chronic kidney disease, chronic obstructive pulmonary disease, osteoporosis, cardiovascular disease, diabetes, chronic hepatitis, end-stage liver disease, lung cancer, and colorectal cancer relative to other adults of the same age, reducing their overall quality of life [3,4]. Geriatric syndromes such as frailty, falls, and functional impairment are also common in people aging with HIV and are experienced at relatively younger ages compared to the general population [5]. Furthermore, people aging with HIV diagnosed earlier in the epidemic received an array of treatment regimens with varying levels of efficacy. There is evidence that the cumulative toxicity of these various treatments over time may have impacted organ functioning [6]. Despite the complexity of multimorbidity, there exists a nationwide shortage of dually trained HIV and aging specialists who are capable of delivering multidisciplinary care to older adults [7]. The clinics where they work also face persistent challenges in obtaining reimbursement for geriatric assessments, creating structural barriers that limit the integration of geriatric care into standard HIV care visits [8].

In addition to medical conditions, people aging with HIV are more likely to be diagnosed with substance use disorders and mental health challenges compared to the general population and experience psychosocial needs related to social isolation, loneliness, stress, and stigma [9]. These experiences may be related to losses through the untimely death of partners, close friends, and community members during the height of the AIDS epidemic, as well as a lack of informal, peer, or familial support and additional discrimination, rejection, or abuse when they seek long-term care via professional care services, such as home care, retirement homes, or skilled nursing facilities [10,11].

While these medical and psychosocial conditions can be managed using tailored treatment guidance, existing screening measures, and other efforts to integrate HIV care into existing geriatric primary health care delivery services, there remains a notable absence of comprehensive, evidence-based programs to support the medical and psychosocial needs of people aging with HIV. A recent review of efforts in the United States and other high-income countries identified several pilot programs designed to bridge this gap [12]. These programs often featured colocation of geriatric and HIV services, as well as comprehensive geriatric screenings delivered in HIV care exams. Although these initiatives have demonstrated feasibility and have suggested potential benefits to clients, their evidence remains preliminary. Importantly, many of the reported programs have since been discontinued, limiting the ability to assess their sustainability, scalability, or long-term outcomes. Consequently, the current evidence base is insufficient to inform the development of standardized care models. Rigorous evaluation using an implementation science (IS) framework can examine promising approaches to address aging-related issues for people with HIV, further building the evidence base of effective interventions to improve health outcomes and quality of life in this population.

### Overview of the Aging With HIV Initiative

To address these identified gaps in comprehensive services for people aging with HIV, the HRSA Ryan White HIV/AIDS Program Part F Special Projects of National Significance (SPNS) program funded the Emerging Interventions to Improve Health Outcomes for People Aging with HIV initiative (also called the Aging with HIV initiative). This initiative supports 10 demonstration sites, an evaluation provider, and a capacity-building provider (CBP) to jointly implement and evaluate emerging interventions to screen and address comorbidities, geriatric conditions, behavioral health, and psychosocial needs of people aging with HIV.

Table 1 lists the demonstration sites of the Aging with HIV initiative, their geographic location, the name of the intervention, the emerging intervention or framework, and a description of their approach. Where applicable, we cited any literature that the demonstration sites used in the development of their interventions. Sites were encouraged to involve patient liaisons as people with lived experience to inform the implementation of their strategy.

**Table 1:** Demonstration site name, location, intervention, and program description.

Location	Intervention name	Program description	Reference
Colorado Health Network, Inc, Denver, Colorado	Integrated Care for Healthy Aging and Navigation of Geriatric Effects (iCHANGE)	Implementing patient-centered, integrated geriatric screening, assessment, coordinated care planning, and management approach based on the geriatric 5Ms (mind, mobility, medications, multicomplexity, and matters most to me) framework.	[13]
Empower U Inc, Miami, Florida	Educating and Empowering People Aging with HIV (E&E program)	Enhancing a “one-stop shop” to provide a suite of services, including dental services, nutritional services, social support groups, and cognitive support groups.	[14,15]
Family Health Centers of San Diego, San Diego, California	Intensive Individualized Care Coordination to Enhance Health and Quality of Life for HIV-Positive Older Adults in San Diego, California (I^2^C^2^)	Comprehensively screening and managing medical and psychosocial comorbidities through improved assessment processes, optimized care coordination, a community advisory board, individualized socialization action planning, and interdisciplinary staff training.	[16,17]
Centro Ararat, Inc, Ponce, Puerto Rico	Platinum Premier Program—integrated care	Implementing a holistic suite of services, including the “friendly hand” of the geriatric health worker, an integrated mental health program, a patient-centered educational and empowerment program, and enhanced health care provider skills in the aging process.	[13]
University of Chicago, Chicago, Illinois	HIV Dementia Champion Training Program—expansion of dementia assessment and management capacity	Expanding dementia assessment and management within care clinics via a staff training program adapted from Scotland’s National Dementia Champions Program and the Dementia Resource Champions program on the South Side of Chicago.	[18]
University of Pittsburgh Medical Center Presbyterian Shadyside, Pittsburgh, Pennsylvania	IMPACT (Improving the 6Ms at Pittsburgh Area Center for Treatment) based on the 5Ms model of care: mind, mobility, multicomplexity, medications, matters most, and modifiable	Implementing an adapted version of the 5Ms framework with an added M (modifiable) to improve patient safety, health outcomes, and care satisfaction through health care provider training, patient screening, and workflow improvements.	[19]
Beth Israel Medical Center, New York, New York	Incorporating a community health worker into a comprehensive program of integrated care for older adults with HIV	Expanding and refining an interdisciplinary pilot program offering a practice-within-practice model to create efficiencies and provide comprehensive geriatric assessments to older adults with HIV.	[8,13,20-22]
Boston Medical Center Corporation, Boston, Massachusetts	HIV-Endurance (HIVE) clinic	Establishing a referral-based, integrated infectious disease and geriatric clinic for patients living with HIV to identify, refine, and assess strategies that comprehensively screen and manage comorbidities, geriatric conditions, behavioral health, and the psychosocial needs of people living with HIV aged 50 years and older.	[8,22]
Wake Forest University Health Sciences, Winston-Salem, North Carolina	Targeting Frailty in Persons Aging with HIV	Implementing geriatric screening assessments for people with HIV aged 50 years and older, focusing on people with frailty or prefrailty by the electronic frailty index. Offering interventions to promote successful aging and combat frailty and prefrailty, including a customized activity and nutrition plan.	[23,24]
Yale University, New Haven, Connecticut	Intervention for collaborative care to assess risk and eliminate polypharmacy, falls, and fragility fractures for people aging with HIV (4F)	Developing a collaborative care model using the 4F framework to manage conditions associated with aging in HIV, improving care delivery, and reducing health disparities.	[25-28]

Health Research, Inc., part of the New York State Department of Health, received the CBP award to support sites through various activities, including one-on-one coaching from implementation specialists. The CBP also plans and hosts a 7-session learning collaborative across the first and second years of the initiative to bring sites together in their coaching groups, share progress on their protocols, facilitate peer-to-peer learning, and develop action plans for the upcoming months.

NORC at the University of Chicago received the evaluation provider award. Incorporating the HRSA HAB IS approach, NORC designed a multicomponent evaluation as an IS hybrid type 2 study with two overarching goals to assess the following:

1. Implementation outcomes, including barriers to and facilitators of implementation, costs, integration into practice, and sustainability
2. Impact of the emerging interventions on client outcomes, including continuum of care outcomes, quality of life, and health care satisfaction

### Purpose

This paper describes 10 demonstration sites integrating HIV and aging-related services, the instruments used to evaluate their activities, the technical assistance (TA) provided for evaluation, and plans to disseminate findings to the broader Ryan White HIV/AIDS Program community and the HIV field. The included sites are distributed nationally and include a variety of settings, from community-based health centers to large academic medical centers, acknowledging that aging needs can vary geographically and culturally and are often relative to the available resources within participating institutions. Clients aged 50 years and older with HIV were eligible for enrollment in the intervention and evaluation. Sample sizes for the evaluation are detailed subsequently.

## Methods

### Overview

NORC will apply 3 complementary IS frameworks to evaluate the Aging with HIV initiative. These are the HAB IS [29]; the reach, effectiveness, adoption, implementation, and maintenance (RE-AIM) [30]; and the Consolidated Framework for Implementation Research (CFIR) [31] frameworks. The HAB IS acknowledges that the uptake and integration of intervention strategies are affected by implementation strategies and broader contextual barriers and facilitators. The HAB IS framework seeks to answer the following core questions:

- Was the intervention strategy successfully implemented in the setting?
- What about the context and environment that shaped its implementation?
- Were the clients successfully engaged in the intervention?
- Were there associated improvements in client outcomes along the HIV care continuum?

The HAB IS calls for integration with other IS frameworks for a more holistic view of implementation outcomes [29]. RE-AIM offers a pragmatic structure to define what constitutes implementation success by assessing the reach and characteristics of participating clients, the degree of organizational and staff adoption, the outcomes of interventions (whether positive, negative, or null), the extent of integration into routine practice, and the sustainability of these outcomes beyond the funding period. The CFIR provides a comprehensive set of constructs across 5 domains—intervention characteristics, inner setting, outer setting, individual characteristics, and process—that enable systematic exploration of the contextual factors shaping implementation.

The combination of these frameworks is uniquely suited to address the gaps identified in aging with HIV interventions, and this combination has been successfully used in other studies. HAB IS provides a solid foundation for testing the intervention and tracking and categorizing the progression of evidence from emerging to evidence-based. RE-AIM defines and measures outcomes from multiple aspects to determine whether those interventions achieve meaningful uptake, effectiveness, and sustainment. CFIR then supplies the theoretical and contextual depth to explain why interventions succeed or fail in particular settings, offering valuable information for replicators to understand potential adaptation. Together, these frameworks allow for the simultaneous testing of implementation strategies, evaluation of client and service outcomes, and systematic identification of contextual determinants, thereby producing actionable knowledge to inform replication and scale-up in diverse care settings.

With a combined framework grounding the evaluation, NORC will design and conduct a hybrid implementation-effectiveness study that dually focuses on implementation processes and implementation, service, and client outcomes [32]. A hybrid study design allows for simultaneous intervention testing and analysis of health data (eg, changes along the HIV care continuum), aligning with the goals of the HAB IS, RE-AIM, and CFIR.

The first objective of NORC’s IS approach is to study rapid implementation using a systematic approach to identify effective emerging interventions, determine their core elements, and disseminate them through multimedia toolkits that are easily accessible and facilitate rapid replication. While there are promising interventions to address aging-related issues in people aging with HIV, there is generally a paucity of research on what is effective. Research that has been conducted typically has small to moderate effects or small sample sizes that limit generalizability. Thus, additional research using the HAB IS framework is critical for strengthening the evidence on effective interventions for people aging with HIV and ultimately improving their health outcomes [33].

The second objective of NORC’s IS approach is assessing client and implementation outcomes, implementation strategies, and barriers and facilitators. The HAB IS model provides a structure for transforming emerging interventions into evidence-informed interventions and, ultimately, into evidence-based interventions [29].

**Figure 1:**
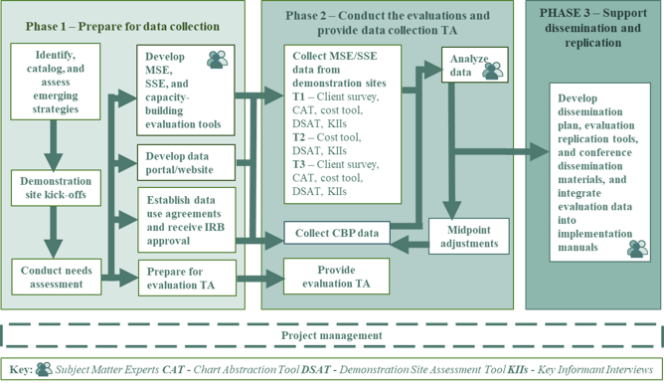
Phases of evaluation. CBP: capacity-building provider; IRB: institutional review board; MSE: multisite evaluation; SSE: specific site evaluation; TA: technical assistance.

NORC selected this combination of frameworks to comprehensively capture and describe the factors that influence the implementation of the emerging interventions selected by the demonstration sites. For example, Figure 1 describes how these frameworks were applied to structure the project in 3 phases: preparing for data collection, conducting the evaluation and providing data collection TA, and supporting dissemination and replication. During each phase, the project is guided by a panel of subject matter experts with experience caring for those aging with HIV, individuals who themselves are aging with HIV, and end users of emerging intervention dissemination materials. The HRSA HAB and demonstration sites provided input on the evaluation plan and instruments throughout their development.

NORC’s mixed methods IS evaluation relies on information collected from demonstration sites and clients to inform 3 different aspects of the evaluation, which will be informed by different data collection instruments:

- Multisite evaluation—Demonstration Site Assessment Tool (DSAT) survey, key informant interviews (KIIs), client survey, cost tools, monthly call notes, and a chart abstraction tool (CAT), which relies on sites to abstract chart data describing key outcomes
- Site-specific evaluation—modules added to the client survey upon the site’s request
- CBP evaluation—DSAT, KIIs, and a quarterly CBP evaluation survey

The data collection structure was initially based on the protocol and lessons learned from the Implementation of Evidence-Based Behavioral Health Models to Improve HIV Health Outcomes for Black Men who have Sex with Men SPNS initiative [34], which used similar instruments. NORC completed the development and selection of these instruments for the Aging with HIV initiative in conjunction with the demonstration sites, taking into consideration the anticipated client and staff burden, as well as expectations from the funder. Efforts to decrease the burden and promote site buy-in included reducing administration frequency and length of instruments, reducing duplication (eg, client Patient Health Questionnaire scores were not collected in both client-facing and CATs), and aligning administration time points for multiple instruments. Sites were required to participate in the evaluation and support data collection according to the terms of their grant.

Data will be collected at 3 main time points over the course of the initiative. The timing within each site will depend on when the site begins implementation. Baseline data collection will occur at or before the implementation begins, midpoint, and end point. The time point for each instrument is described subsequently.

### Needs Assessment

NORC began the evaluation by reviewing all submitted materials to establish the maturity of the intervention and any previous evaluations. NORC also assessed each site’s readiness to conduct evaluations based on the plans submitted to HRSA. NORC compiled the results of the needs assessment and met with HRSA to discuss potential barriers to and facilitators of the evaluation, as well as any potential adjustments needed to the evaluation plan.

### DSAT Survey

The DSAT documents quantitative, site-level implementation outcomes that are mapped to the overarching research questions. Key domains were built on the RE-AIM and CFIR domains and included staffing, client enrollment in the intervention, client needs for health care and social services, partnerships, organizational culture, leadership, factors influencing implementation, funding sources, and sustainability. NORC encourages sites to meet as a team to complete 1 DSAT response per site at baseline, midpoint, and follow-up to assess how implementation changes over time.

### KII Data

KIIs are particularly useful in two contexts: (1) taking an in-depth look at a particular topic that emerged through a TA interaction, and (2) conducting detailed reviews with implementation sites and the CBP to assess emerging quantitative results in a structured way to ensure correct understanding and add richness to the findings. NORC will take detailed notes and conduct rapid thematic analysis for site-specific and cross-site themes. Potential respondents for KIIs include principal investigators, project managers, and patient liaisons, with at least 1 respondent per site at baseline, midpoint, and follow-up.

### Client Survey

Outcomes related to exposure to interventions, experience with health care services, and quality of life (EQ-5D) must be measured with data from clients. We developed a brief client survey that balances the need for client data while minimizing the burden on intervention clients. Where such measures existed, we included validated measures that have been applied in HIV care settings, including other SPNS projects, as shown in Table 2. The Office of Management and Budget updated the standards for race and ethnicity measurements after baseline, which will be updated for follow-up.

**Table 2:** Survey domains from validated scales and similar projects.

Item or domain	Source
Race and ethnicity (at baseline)	OMB^a^ [35]
Race and ethnicity (at follow-up)	OMB [36]
Quality of life	EQ-5D [37]
Unmet needs for mental health services	Black MSM^b^ SPNS^c^ initiative [34]
Care satisfaction	Black MSM SPNS initiative [34]
Cultural humility	ESCALATE SPNS initiative [38]
Loneliness	University of California, Los Angeles Loneliness Scale [39]
Acuity	American Community Survey [40]
Frailty	FRAIL^d^ scale [41]

^a^OMB: Office of Management and Budget.

^b^MSM: men who have sex with men.

^c^SPNS: Special Projects of National Significance.

^d^FRAIL: fatigue, resistance, ambulation, illness, and loss of weight.

We offered each site multiple approaches to recruit clients and complete the self-administered survey electronically or on paper, with a preference for electronic administration to minimize the staff time needed for data entry. We provided each site with a QR code and survey link that they could share via email, clinic messaging portal (eg, MyChart), or signage. TA coaches asked sites about clients’ needs for adaptation, particularly for translation into other languages. As a result, versions of the survey are available in English, Spanish, Haitian Creole, and Arabic, with translations provided by a certified translation company that is tailored to the specific dialects used by demonstration site clients. Adaptations of the paper survey include these translations and a low-vision, high-contrast version in English. These data will be collected at the baseline and at the project conclusion.

NORC offered sites the option to add site-specific questions to the client survey to measure outcomes particular to each emerging strategy. Given that the client survey is comprehensive and already includes many of the site-specific outcomes, only 2 sites requested additional data collection. These include questions on relationship satisfaction, self-care satisfaction, substance use, food insecurity, servings of fruit and vegetables, and exercise frequency.

NORC calculated site-specific baseline enrollment targets based on the number of clients anticipated to be enrolled in each site’s intervention, given their capacity for the pilot nature of the interventions and in conversation with the funder. We then accounted for refusals and losses to follow-up to arrive at a site-specific sample capable of producing estimates with an error margin of less than 5% for reasonable effect sizes. The baseline enrollment targets at each site ranged from 30 to 150 participants, with an average of 66 participants per site.

On the basis of the early feedback received from implementation sites to put survey data into action for care improvement, we will compile all completed surveys weekly and share them with sites via a secure platform for their review and follow-up. Given that certain questions can be used in a clinical setting as screening tools, we will calculate each client’s score and apply stoplight-style color coding. This will facilitate the clinical staff’s review of the client responses and rapid response. We will also highlight any surveys that remain incomplete for 2 weekly downloads and ask the site to follow up with the client.

### Cost Tool

Total intervention costs will be assessed by combining the total HRSA-provided funding with other funding sources and in-kind or volunteer supports. Key domains include staffing, intervention-specific space, intervention materials, other direct costs, and indirect costs. We will use this information to calculate the total intervention cost and cost per client served. Should the intervention result in meaningful changes to the client’s quality of life, we will assess quality-adjusted life years and cost-effectiveness per US $100,000 saved in societal costs. To assist potential replication sites in estimating the cost of each emerging strategy, we will qualitatively assess the percentage of funding spent on clinical services and other replicable components (eg, client engagement and community partnership) relative to HRSA HAB SPNS initiative requirements (eg, grants administration, learning sessions, protocol development, and other dissemination tools) that would not pertain to potential replication sites funded by means other than a SPNS initiative.

### Monthly Call Notes

We will evaluate TA calls with each demonstration site on a monthly basis in collaboration with the CBP and the site’s HRSA project officer. Each demonstration site will be asked to complete a monthly call form at least 5 days before the joint monitoring call, which will allow time for HRSA, NORC, and the CBP to review the information and prepare for the discussion. During the calls, the demonstration sites will elaborate on the information they provide in the monthly call form related to implementation strategies, activities, ongoing challenges, mitigation strategies, successes, and lessons learned related to implementing their emerging interventions. The HRSA, NORC, and CBP will have the opportunity to ask follow-up questions. During the discussion, NORC evaluation TA coaches will update and annotate the monthly call form so that it is the document of record for sites’ activities. Following the call, NORC team will review and finalize the notes and updates in the monthly call form. The evaluation team will use the sites’ monthly call forms and DSAT data to tailor the KII guide to each site before conducting the interviews. The monthly call form will also provide a record for noting any client who withdraws, as well as enrollments in the intervention after baseline client data collection has been completed.

### CAT Data

Demonstration sites will be asked to abstract client data from their charts or electronic medical record systems and submit this via an Aging with HIV CAT. Clients will be asked to consent to having their selected clinic-level electronic medical record data shared with NORC when they consent to the client survey. This will include data on client screenings, use of services (and what type of services), engagement in care (eg, linkage to care and retention in care), referrals, antiretroviral treatment prescriptions, and viral load. They will provide these data at the same time points as the client survey, that is, baseline and end point.

The data will be used to track the influence of the emerging interventions being implemented at each site on screening and referral of geriatric conditions, as well as typical HIV-related outcomes. NORC will provide each site with a preassigned list of client identifiers. Sites will then assign clients a unique identifier from this at enrollment that will be tied to their chart data, enabling it to be linked to their client survey responses.

The CAT will also collect information on each site’s anticipated outcomes to provide data for the site-specific evaluation. This will include data on diagnoses of neurological functioning (which cannot be reliably self-reported), weight, hypertension, cholesterol, referrals for services (including nutrition, oral health, mental health, memory care, physical therapy, and substance use treatment), unplanned hospitalizations, and emergency department visits. In addition, the CAT will collect information on client outcomes related to common HIV and aging comorbidities, geriatric conditions, behavioral health, and psychosocial needs. This will enable us to assess whether the emerging interventions used by the sites improve outcomes for people aging with HIV (goal 2).

As noted earlier, demonstration sites that collect data on sensitive issues related to client safety and require timely follow-up and mandatory reporting will be asked to share these data via a custom tab in the CAT. They will submit deidentified client data through a secure, password-protected, web-based portal and a data repository built in Microsoft SharePoint.

### CBP Survey

The CBP evaluation survey is a brief, web-based survey that we programmed in Qualtrics and will be administered to sites’ intervention teams on a quarterly basis throughout the preimplementation and implementation phases. The CBP evaluation survey will ask intervention teams to evaluate capacity-building activities that CBP has offered or facilitated with demonstration sites during the 3-month look-back period, such as the learning sessions, affinity group meetings, one-on-one coaching, and peer learning meetings. It will also capture the dosage, perceptions, and needs of implementation teams as related to the capacity-building activities they have engaged in over the previous quarter. We will provide the CBP and HRSA with a topline report on the findings to inform CBP’s real-time quality improvement efforts.

### Evaluation TA

We will offer TA to sites to support the completion of the abovementioned instruments. We will assign a staff member to be a single point of contact with each site and attend the monthly monitoring calls described earlier. We will also offer a dedicated inbox to receive and track TA requests that arise between calls. We will prepare an evaluation TA checklist to be completed along with the site during an in-person coaching visit. TA requests requiring more than minimal effort, such as responding to an email, will be documented for future analyses. In the first 18 months of the project, there were fewer than 20 such requests.

### Ethical Considerations

We will develop study materials and submit them for clearance through NORC’s institutional review board (IRB). NORC will implement a business associate agreement with each demonstration site (and the CBP, as appropriate) to facilitate the sharing of datasets for their own individual analyses. NORC will not begin data collection until we receive appropriate human participants’ approval and have the necessary business associate agreements in place with the demonstration sites.

We will offer sites the opportunity to rely on NORC’s IRB to facilitate obtaining the appropriate clearances for the collection of client data. For those sites that choose to use their own IRBs, we will provide instruments, consent forms, and protocols to support their applications. A total of 6 sites relied upon NORC’s IRB. However, NORC’s IRB will review and approve the organizational-level data collection instruments and methods for all sites.

NORC operates its own IRB, which is registered with the US Department of Health and Human Services Office of Human Research Protections (Federalwide Assurance 00000142). NORC’s IRB follows a formal process for examining all research projects to ensure human participants’ protections and minimize respondent burden. NORC will develop the IRB protocol to address human participants’ protections that includes all study materials, such as recruitment materials, informed consent statements, and data collection instruments. This will include applying for a waiver of written consent for NORC to avoid collecting client names and to mitigate potential concerns of clients who may need to rely on caregivers to consent. Instead, as described in the informed consent, participants will be considered to have consented to the survey after reading the informed consent and clicking next (electronic version) or continuing to the survey questions (paper version). The informed consent will include instructions on how to withdraw consent in the future. NORC will provide TA to sites on ethical data collection standards and report any deviation of the data collection protocol to the IRB immediately. HRSA awarded a certificate of confidentiality automatically as part of the grant-making process, which covers all data collected under the evaluation. NORC did not offer compensation to respondents.

### Analysis Plan

To ensure harmonization across the various data systems at the demonstration sites, we will ask a data liaison at each site to enter the data into the standardized tools described earlier. We will offer an evaluation TA to the data liaisons to train them in the tools and completion instructions, and respond to any issues or questions that arise. We will also conduct quality checks of the data as they are submitted, and spot-check a random sample of 10% of the data points during an in-person audit and coaching visit.

Once received, we will clean the data according to accepted data quality standards. We will then analyze and report data on an ongoing basis and, in collaboration with sites and HRSA, will provide data to inform intervention adjustments. Key methods will include unadjusted change in outcomes pre and postintervention and paired 2-tailed *t* tests for continuous outcomes. Because clients are nested within sites, we will also conduct sensitivity analyses using mixed-effects models with random intercepts for each site to account for clustering. Analyses will be conducted using complete case data, with sensitivity checks to evaluate the impact of missingness. Because client survey data are collected during routine program activities, the anticipated rate of missingness is relatively low. As findings emerge, we will review these in collaboration with sites in “data parties,” to which the CBP and HRSA will be invited to provide input and contextualization [38]. NORC will make results available to all sites in the form of a master slide deck available on the internal SharePoint site. Sites requiring additional analyses will be able to make requests for NORC’s assistance through their TA coach.

### Dissemination Planning

Consistent with the IS approach, we will plan for rapid dissemination from the start of the initiative and focus as much on the barriers to and facilitators of implementation as on the resulting client outcomes. The evaluation findings will be part of an initiative-wide effort on dissemination led in partnership with HRSA, the CBP, and the demonstration sites. Expected venues for dissemination include TargetHIV.org, the training and TA repository for the Ryan White HIV/AIDS Program–funded programs, peer-reviewed publications, and professional conferences.

## Results

Organizational-level data collection began with monthly call forms starting in April 2023, followed by the administration of the DSAT and then KIIs. The first site began recruitment for their intervention in May 2023 and enrollment in the evaluation in the same month. In all the sites, enrollment was underway by September 2023, and 626 clients were enrolled by December 2023 when baseline data collection ended. Evaluation enrollment and follow-up client data collection is expected to continue through December 2024. Key recruitment challenges included concerns about sharing personal information and challenges encountered in completing the online survey. Facilitators included showing clients how to use a QR code, offering paper surveys as an alternative, and providing incentives for completion. As of February 2024, 136 clients had withdrawn. The most common reason for withdrawing was sites losing contact, although a few clients withdrew their consent. Data submissions have met standards for quality and completeness after provision of evaluation TA, and no data issues were identified in on-site audits. Analysis of baseline data is currently underway and will inform midpoint and end point data collection. Comprehensive findings are expected in late 2025.

## Discussion

### Anticipated Findings

This paper outlines a protocol for the evaluation of emerging strategies to support people aging with HIV designed to assess key barriers to, challenges to, and facilitators of implementation, along with their potential effect on clients. Currently, HIV care in the United States lacks integration with geriatric care, and many of the pilot programs in this field have not been sustained or replicated [12]. Our nationwide sample of diverse care settings implementing a variety of aging-related supports will offer rich information analyzed with an IS lens to ensure that the findings are applicable to an array of clinical settings in the United States. We expect that the results of our evaluation will advance HIV clinical care nationally by contributing to the evidence base of efforts to integrate HIV care with aging-related care. The interventions we evaluate include general geriatric interventions adapted for people with HIV to best meet the needs of the people served by the various implementation sites. Consequently, providers of health care for people aging with HIV may likely identify relevant aspects that could be applied to their own practice.

### Limitations

To the best of our knowledge, our study is one of the first of its kind to evaluate various aging-support interventions for people with HIV in different clinical and community settings nationally through primary data collection, but it is not without limitations. First, our sites include a variety of practices from large, academic medical centers to small, community-based clinics, yet all received grant funding for this initiative; therefore, our findings may not be representative of other practice models and health care providers attempting to do this work without additional resources. We will seek to highlight the approaches that show the most promise for implementation in diverse settings, including lower-resourced settings, such as the community health centers included in the initiative. This could include making available screening materials and electronic health record templates used in this initiative. Second, clients participating in the evaluation were largely already enrolled and retained in care by the sites in which they received the integrated services, which may introduce bias into our measurement of satisfaction and retention in care. The number of clients enrolled at each site depended largely on the nature of the intervention being piloted, and subgroup analysis of final data may be limited if participation is low. Finally, given the amount of time necessary to stand up new interventions within a defined initiative period, we may have insufficient time to detect longer-term client outcomes and their economic impact. We will acknowledge these limitations (eg, those related to sample size, temporality, and potential sources of survey error) in the dissemination of the empirical findings.

Despite these limitations, we are confident that our evaluation will yield important insights to inform implementation by other HIV care providers. Consistent with our IS approach, null and negative results are just as important for dissemination as positive results. Should the interventions fail to demonstrate measurable improvements in client health, our implementation information could help explain why that may have happened or what future replicators may need to do differently. We intend to disseminate findings through a peer-reviewed article describing the high-level findings from all demonstration sites, along with topic-specific articles (eg, approaches to addressing frailty) written in collaboration with demonstration sites. To assist practitioners, we will also develop publicly available fact sheets and case studies designed for rapid uptake by health care providers and other interest holders. The resulting dissemination products will provide practical contributions to building the evidence base for integrating an aging perspective into HIV care and will help other HIV care providers identify the strategy that best supports their clients aged 50 years and older.

## Data Availability

The datasets generated or analyzed during this study are not publicly available due to their nature as protected health information from vulnerable populations and corresponding restrictions among participating institutions.

## Abbreviations

CAT: chart abstraction tool
CBP: capacity-building provider
CFIR: Consolidated Framework for Implementation Research
DSAT: Demonstration Site Assessment Tool
HAB: HIV/AIDS Bureau
HRSA: Health Resources and Services Administration
IRB: institutional review board
IS: implementation science
KII: key informant interview
RE-AIM: reach, effectiveness, adoption, implementation, and maintenance
SPNS: Special Projects of National Significance
TA: technical assistance

## References

[1] (2024). HIV and older people. Department of Health and Human Services.

[2] (2023). Ryan White HIV/AIDS program annual data report. Health Resources and Services Administration.

[3] Turrini G, Chan SS, Klein PW, Cohen SM, Dempsey A, Hauck H, Cheever LW, Chappel AR (2020). Assessing the health status and mortality of older people over 65 with HIV. PLoS One.

[4] Friedman EE, Duffus WA (2016). Chronic health conditions in Medicare beneficiaries 65 years old, and older with HIV infection. AIDS.

[5] Greene M, Covinsky KE, Valcour V, Miao Y, Madamba J, Lampiris H, Cenzer IS, Martin J, Deeks SG (2015). Geriatric syndromes in older HIV-infected adults. J Acquir Immune Defic Syndr.

[6] Margolis AM, Heverling H, Pham PA, Stolbach A (2014). A review of the toxicity of HIV medications. J Med Toxicol.

[7] Norberg A, Nelson J, Lin H, Lazo E, Stanislaus D, Chu C, Bolduc P (2024). A forecast of the HIV clinician workforce need in the United States: results of a quantitative national survey. J Assoc Nurses AIDS Care.

[8] Davis AJ, Greene M, Siegler E, Fitch KV, Schmalzle SA, Krain A, Vera JH, Boffito M, Falutz J, Erlandson KM (2022). Strengths and challenges of various models of geriatric consultation for older adults living with human immunodeficiency virus. Clin Infect Dis.

[9] Fekete EM, Williams SL, Skinta MD (2018). Internalised HIV-stigma, loneliness, depressive symptoms and sleep quality in people living with HIV. Psychol Health.

[10] Bhochhibhoya A, Harrison S, Yonce S, Friedman DB, Ghimire PS, Li X (2021). A systematic review of psychosocial interventions for older adults living with HIV. AIDS Care.

[11] Brennan-Ing M, Seidel L, Karpiak SE (2017). Social support systems and social network characteristics of older adults with HIV. Interdiscip Top Gerontol Geriatr.

[12] Dunville R, Greene M (2025). Innovative models of care supporting people aging with HIV. Curr Opin HIV AIDS.

[13] Tinetti M, Huang A, Molnar F (2017). The geriatrics 5M's: a new way of communicating what we do. J Am Geriatr Soc.

[14] (2025). Evidence of NCQA PCMH effectiveness. National Committee for Quality Assurance.

[15] O'Dell ML (2016). What is a patient-centered medical home?. Mo Med.

[16] (2019). Intensive care coordination for children and youth with complex mental and substance use disorders: state and community profiles. Substance Abuse and Mental Health Services Administration.

[17] Garcia ME, Uratsu CS, Sandoval-Perry J, Grant RW (2018). Which complex patients should be referred for intensive care management? A mixed-methods analysis. J Gen Intern Med.

[18] Banks P, Waugh A, Henderson J, Sharp B, Brown M, Oliver J, Marland G (2014). Enriching the care of patients with dementia in acute settings? The Dementia Champions Programme in Scotland. Dementia (London).

[19] Erlandson KM, Karris MY (2019). HIV and aging: reconsidering the approach to management of comorbidities. Infect Dis Clin North Am.

[20] Parker SG, McCue P, Phelps K, McCleod A, Arora S, Nockels K, Kennedy S, Roberts H, Conroy S (2018). What is comprehensive geriatric assessment (CGA)? An umbrella review. Age Ageing.

[21] Rajabiun S, Falkenberry H, Downes A, Frye A, Davich J, Wilkinson G, Baughman A, Bowers-Sword R, Campos Rojo M, Allen C, Drainoni ML, Bachman S, Sprague Martinez M (2020). A guide to implementing a community health worker (CHW) program in the context of HIV care. Boston University.

[22] Levett T, Alford K, Roberts J, Adler Z, Wright J, Vera JH (2020). Evaluation of a combined HIV and geriatrics clinic for older people living with HIV: the Silver Clinic in Brighton, UK. Geriatrics (Basel).

[23] Erlandson KM, MaWhinney S, Wilson M, Gross L, McCandless SA, Campbell TB, Kohrt WM, Schwartz R, Brown TT, Jankowski CM (2018). Physical function improvements with moderate or high-intensity exercise among older adults with or without HIV infection. AIDS.

[24] Stuck AE, Siu AL, Wieland GD, Adams J, Rubenstein LZ (1993). Comprehensive geriatric assessment: a meta-analysis of controlled trials. Lancet.

[25] Guaraldi G, Marcotullio S, Maserati R, Gargiulo M, Milic J, Franconi I, Chirianni A, Andreoni M, Galli M, Lazzarin A, D'Arminio Monforte A, Di Perri G, Perno CF, Puoti M, Vella S, Di Biagio A, Maia L, Mussi C, Cesari M, Antinori A (2019). The management of geriatric and frail HIV patients. A 2017 update from the Italian guidelines for the use of antiretroviral agents and the diagnostic clinical management of HIV-1 infected persons. J Frailty Aging.

[26] Livio F, Marzolini C (2019). Prescribing issues in older adults living with HIV: thinking beyond drug-drug interactions with antiretroviral drugs. Ther Adv Drug Saf.

[27] American Geriatrics Society 2015 Beers Criteria Update Expert Panel (2015). American Geriatrics Society 2015 updated Beers Criteria for potentially inappropriate medication use in older adults. J Am Geriatr Soc.

[28] Brown TT, Hoy J, Borderi M, Guaraldi G, Renjifo B, Vescini F, Yin MT, Powderly WG (2015). Recommendations for evaluation and management of bone disease in HIV. Clin Infect Dis.

[29] Psihopaidas D, Cohen SM, West T, Avery L, Dempsey A, Brown K, Heath C, Cajina A, Phillips H, Young S, Stubbs-Smith A, Cheever LW (2020). Implementation science and the Health Resources and Services Administration's Ryan White HIV/AIDS Program's work towards ending the HIV epidemic in the United States. PLoS Med.

[30] Glasgow RE, Vogt TM, Boles SM (1999). Evaluating the public health impact of health promotion interventions: the RE-AIM framework. Am J Public Health.

[31] Kirk MA, Kelley C, Yankey N, Birken SA, Abadie B, Damschroder L (2016). A systematic review of the use of the Consolidated Framework for Implementation Research. Implement Sci.

[32] Curran GM, Bauer M, Mittman B, Pyne JM, Stetler C (2012). Effectiveness-implementation hybrid designs: combining elements of clinical effectiveness and implementation research to enhance public health impact. Med Care.

[33] (2010). National HIV/AIDS strategy for the United States. The White House Office of National AIDS Policy.

[34] Hodge SE, Johnson-Turbes A, Flemming SS, Passero M, Tinsley M, Iheanyichukwu T, Black MSM Initiative Study Group (2022). Implementation of evidence-informed behavioral health models to improve HIV health outcomes for black men who have sex with men (Black MSM Initiative): protocol for program evaluation. JMIR Res Protoc.

[35] (1997). Revisions to the standards for the classification of federal data on race and ethnicity. The White House Office of Management and Budget.

[36] Orvis K (2024). OMB publishes revisions to Statistical Policy Directive No. 15: standards for maintaining, collecting, and presenting federal data on race and ethnicity. The White House.

[37] Devlin P, Parkin D, Janssen B (2020). An introduction to EQ-5D instruments and their applications. Methods for Analysing and Reporting EQ-5D Data.

[38] Guidry JA (2022). ESCALATE: ending stigma through collaboration and lifting all to empowerment – implementation science evaluation. National Ryan White Conference on HIV Care & Treatment.

[39] The UCLA 3-Item Loneliness Scale. Measuring your impact on loneliness in later life.

[40] (2023). American Community Survey: Methodology. United States Census Bureau.

[41] Woo J, Yu R, Wong M, Yeung F, Wong M, Lum C (2015). Frailty screening in the community using the FRAIL scale. J Am Med Dir Assoc.

